# EXCURSIONS INTO HEALTHCARE SYSTEMS TO HELP INFORMAL CAREGIVERS MANAGE DEMENTIA

**DOI:** 10.1093/geroni/igac059.1281

**Published:** 2022-12-20

**Authors:** Richard Fortinsky, Christopher Callahan

## Abstract

With the establishment in 2019 of the National Institute on Aging-funded IMPACT Collaboratory, the era of designing and implementing pragmatic clinical trials in partnership with health care systems to improve care and health-related outcomes for people living with dementia (PLWD) is well underway. An important focus for Collaboratory-funded work involves targeting and engaging family and other informal caregivers of PLWD across health care system settings. In this Symposium, we feature three Collaboratory-funded investigators who are partnering with health care systems to determine how to engage informal caregivers most pragmatically, with the goals of improving caregivers’ capacity to manage dementia and provide sustained input into the clinical care of PLWD. Symposium presenters Quincy Samus and Hillary Lum are the first two Collaboratory-funded Health Care System Scholars and Richard Fortinsky is a pilot study awardee. Dr. Samus will present on her experiences and lessons learned in her multiple efforts to adapt and embed the MIND at Home family-focused care management model into health care systems, including her current work with a large managed care organization. Dr. Lum will explain ongoing capacity-building stakeholder engagement activities at a large academic health system in preparation for pragmatic clinical trials for PLWD and their caregivers. Dr. Fortinsky will present on experiences and lessons learned from ongoing efforts to identify caregivers of PLWD to join caregiver support programs, and store caregiver data electronically, in two health system outpatient care settings. Dr. Christopher Callahan, Leader of the Training Core for the Collaboratory, will serve as Symposium Discussant.

